# Supplementary material for: Genome-wide association analysis of type II resistance to Fusarium head blight in common wheat
Source: PeerJ. 2023 Sep 21;11:e15906. doi: 10.7717/peerj.15906 (PMC10518165; doi:10.7717/peerj.15906)
Supplement: Table S1 — a E1, E2 and E3 were same as the Table 1. b Chromosome. [file peerj-11-15906-s001.docx]

**Supplementary Table 1** SNP markers significantly associated with FHB resistance in three environments

| Env^a^ | Marker | Chr^b^ | Position  (cM) | Disease spikelets rate | | Disease spike rachis rate | |
| --- | --- | --- | --- | --- | --- | --- | --- |
|  |  |  |  | *P*-value | PVE(%) | *P*-value | PVE(%) |
| E1 | *BS00095100_51* | 1A | 71 |  |  | 7.16E-04 | 6.01 |
|  | *GENE-0071_115* | 1A | 71 |  |  | 3.22E-04 | 6.82 |
|  | *BS00087544_51* | 1B | 107 | 9.00E-04 | 5.66 |  |  |
|  | *Excalibur_c91176_326* | 2A | 150 | 9.78E-04 | 5.55 |  |  |
|  | *TA003865-0902* | 2B | 32 | 8.78E-04 | 5.66 |  |  |
|  | *wsnp_BE489901D_Ta_2_1* | 2D | 131 | 9.21E-04 | 5.61 |  |  |
|  | *Tdurum_contig19920_463* | 4A | 109 |  |  | 9.77E-04 | 5.70 |
|  | *D_contig74317_533* | 5D | 21 | 2.01E-04 | 7.11 | 1.65E-04 | 7.51 |
|  | *wsnp_BE498985A_Ta_2_1* | 7B | 103 | 5.64E-04 | 6.11 |  |  |
|  | *wsnp_BE605194B_Ta_2_1* | 7B | 103 | 5.64E-04 | 6.11 |  |  |
|  | *wsnp_BE605194B_Ta_2_7* | 7B | 103 | 7.08E-04 | 5.87 |  |  |
|  | *GENE-4996_592* | 7B | 103 | 7.08E-04 | 5.87 |  |  |
|  | *Tdurum_contig10677_529* | 7B | 103 | 5.64E-04 | 6.11 |  |  |
|  | *BS00066342_51* | 7B | 155 | 1.15E-04 | 7.82 |  |  |
| E2 | *Kukri_c14239_1995* | 1B | 86 | 9.70E-05 | 8.46 | 1.28E-04 | 8.11 |
|  | *Excalibur_c11587_340* | 2B | 104 | 7.64E-04 | 6.14 |  |  |
|  | *BS00012071_51* | 2B | 107 | 6.22E-04 | 6.36 |  |  |
|  | *Excalibur_c74466_344* | 2B | 107 | 5.81E-04 | 6.45 |  |  |
|  | *IAAV3305* | 2B | 91 |  |  | 3.91E-04 | 6.84 |
|  | *Kukri_rep_c109397_59* | 5B | 6 |  |  | 9.60E-04 | 5.90 |
|  | *Excalibur_c3948_1315* | 5B | 20 |  |  | 3.44E-04 | 6.98 |
|  | *Kukri_c2514_490* | 5B | 20 |  |  | 3.44E-04 | 6.98 |
|  | *Kukri_c2514_583* | 5B | 20 |  |  | 3.44E-04 | 6.98 |
|  | *Tdurum_contig25432_1020* | 5B | 20 |  |  | 3.44E-04 | 6.98 |
|  | *Tdurum_contig25432_1218* | 5B | 20 |  |  | 3.44E-04 | 6.98 |
|  | *Tdurum_contig25432_1377* | 5B | 20 |  |  | 3.44E-04 | 6.98 |
|  | *BobWhite_c3073_1156* | 6A | 12 |  |  | 6.69E-04 | 6.28 |
|  | *wsnp_Ku_c39334_47795350* | 6A | 13 |  |  | 9.33E-04 | 5.93 |
|  | *wsnp_Ku_c39334_47795461* | 6A | 13 |  |  | 4.68E-04 | 6.66 |
|  | *BS00090253_51* | 6A | 13 |  |  | 6.96E-04 | 6.32 |
|  | *Excalibur_c431_1130* | 6A | 13 |  |  | 4.69E-04 | 6.65 |
|  | *RAC875_c68978_220* | 6A | 13 |  |  | 4.69E-04 | 6.65 |
|  | *TA005787-0140* | 6A | 13 |  |  | 4.69E-04 | 6.65 |
|  | *IAAV9150* | 6A | 17 |  |  | 1.60E-04 | 7.82 |
|  | *Kukri_c3009_1702* | 6A | 17 |  |  | 1.60E-04 | 7.82 |
|  | *RFL_Contig5170_330* | 6A | 17 |  |  | 3.42E-04 | 6.98 |
|  | *Tdurum_contig63703_1143* | 6A | 17 |  |  | 2.37E-04 | 7.38 |
|  | *wsnp_Ra_c3996_7334169* | 6A | 17 |  |  | 2.36E-04 | 7.37 |
|  | *Excalibur_c20597_509* | 6A | 17 |  |  | 1.77E-04 | 7.69 |
|  | *wsnp_Ex_c7002_12063325* | 6A | 131 |  |  | 8.98E-04 | 5.98 |
|  | *wsnp_Ex_c7002_12063380* | 6A | 131 |  |  | 8.98E-04 | 5.98 |
|  | *Excalibur_c7002_314* | 6A | 131 |  |  | 8.98E-04 | 5.98 |
|  | *CAP11_c3666_426* | 7A | 212 |  |  | 8.94E-04 | 7.04 |
|  | *BS00025278_51* | 7B | 92 | 5.28E-04 | 6.56 |  |  |
|  | *BS00025286_51* | 7B | 92 | 2.84E-05 | 11.20 | 8.77E-04 | 7.07 |
| E3 | *Kukri_c7902_438* | 1B | 64 | 4.11E-04 | 6.33 |  |  |
|  | *Excalibur_c27675_1815* | 1B | 64 | 6.18E-04 | 5.93 |  |  |
|  | *CAP7_c1241_128* | 1B | 65 | 6.40E-04 | 5.90 |  |  |
|  | *tplb0032i02_1435* | 2A | 25 | 5.75E-04 | 6.00 |  |  |
|  | *wsnp_Ra_c21104_30458226* | 2A | 25 | 5.75E-04 | 6.00 | 2.79E-04 | 6.73 |
|  | *Ra_c510_171* | 2A | 25 |  |  | 6.75E-04 | 6.40 |
|  | *RAC875_c510_923* | 2A | 25 |  |  | 7.01E-04 | 6.36 |
|  | *tplb0032i02_1435* | 2A | 25 |  |  | 2.79E-04 | 6.73 |
|  | *Tdurum_contig76595_208* | 2A | 26 | 2.46E-04 | 7.52 |  |  |
|  | *BS00068050_51* | 2A | 48 | 2.20E-04 | 6.94 |  |  |
|  | *BS00022242_51* | 3B | 54 | 5.09E-04 | 6.12 |  |  |
|  | *Kukri_c7087_896* | 3B | 72 | 8.76E-05 | 7.86 | 2.34E-04 | 6.91 |
|  | *Excalibur_c25678_337* | 3B | 72 | 3.71E-04 | 6.43 | 9.79E-04 | 5.51 |
|  | *RAC875_c35801_905* | 3D | 70 | 3.72E-05 | 8.72 | 5.35E-05 | 8.39 |
|  | *RAC875_c8662_140* | 3D | 143 | 6.18E-04 | 5.93 |  |  |
|  | *CAP7_c4800_276* | 5A | 83 | 2.20E-04 | 6.94 |  |  |
|  | *IAAV8455* | 5B | 151 | 4.29E-04 | 6.29 |  |  |
|  | *BS00099729_51* | 5B | 184 | 7.61E-04 | 5.73 | 9.75E-04 | 5.51 |
|  | *RAC875_c68525_284* | 6B | 77 | 1.57E-04 | 7.27 | 3.30E-04 | 6.57 |
|  | *RAC875_c18043_411* | 7B |  |  | 6.99 | 2.35E-04 | 6.90 |
|  | *Kukri_c4143_1055* | 7B |  |  | 6.96 | 2.49E-04 | 6.85 |

**^a^** E1, E2 and E3 were same as the Table 1. **^b^** Chromosome.

Genetic mapping reference from: Wang et al. 2014
