# Supplementary material for: Genome-wide association analysis of type II resistance to Fusarium head blight in common wheat
Source: PeerJ. 2023 Sep 21;11:e15906. doi: 10.7717/peerj.15906 (PMC10518165; doi:10.7717/peerj.15906)
Supplement: Table S2 [file peerj-11-15906-s002.docx]

**Supplementary Table 2** Physical positions of SNP markers significantly associated with FHB resistance

| SNP marker | Physical position | SNP marker | Physical position |
| --- | --- | --- | --- |
| *BS00095100_51* | chr1A:308572388-308572288(Strand=Plus/Minus) | *IAAV9150* | chr6A:6733144 -6733015(Strand=Plus/Minus) |
| *GENE-0071_115* | chr1A:3553372111-355337111(Strand=Plus/Minus) | *Kukri_c3009_1702* | chr6A:6733065-6733144(Strand=Plus/Plus) |
| *BS00087544_51* | No | *RFL_Contig5170_330* | chr6A:6728201-6728294(Strand=Plus/Plus) |
| *Excalibur_c91176_326* | chr2A:758923336-758923244(Strand=Plus/Minus) | *Tdurum_contig63703_1143* | chr6A:6733524-6733444(Strand=Plus/Minus) |
| *TA003865-0902* | chr2B:20075961-20076014(Strand=Plus/Plus) | *wsnp_Ra_c3996_7334169* | chr6A:6734003-6733803(Strand=Plus/Minus) |
| *wsnp_BE489901D_Ta_2_1* | chr2D:637651044-637651164(Strand=Plus/Plus) | *Excalibur_c20597_509* | chr6A:6725171-6725071(Strand=Plus/Minus) |
| *Tdurum_contig19920_463* | chr4A:659014917-659015017(Strand=Plus/Plus) | *wsnp_Ex_c7002_12063325* | chr6A:601473016-601473216(Strand=Plus/Plus) |
| *D_contig74317_533* | chr5D:5587472-5587720(Strand=Plus/Plus) | *wsnp_Ex_c7002_12063380* | chr6A:601473071-601473237(Strand=Plus/Plus) |
| *wsnp_BE498985A_Ta_2_1* | chr7B:657933278-657933379(Strand=Plus/Plus) | *Excalibur_c7002_314* | chr6A:601472919-601473019(Strand=Plus/Plus) |
| *wsnp_BE605194B_Ta_2_1* | chr7B:657872739-657872619(Strand=Plus/Minus) | *CAP11_c3666_426* | chr7A:708624850-708624750(Strand=Plus/Minus) |
| *wsnp_BE605194B_Ta_2_7* | chr7B:657871754-657871634(Strand=Plus/Minus) | *BS00025278_51* | chr7B:636507892-636507992(Strand=Plus/Plus) |
| *GENE-4996_592* | chr7B:657871574-657871474(Strand=Plus/Minus) | *BS00025286_51* | chr7B:636508126-636508226(Strand=Plus/Plus) |
| *Tdurum_contig10677_529* | chr7B:657933369-657933269(Strand=Plus/Minus) | *RAC875_c23743_552* | chr1B:532399942-532400009(Strand=Plus/Plus) |
| *BS00066342_51* | chr7B:725735700-725735800(Strand=Plus/Plus) | *Kukri_c7902_438* | chr1B:327355988-327355888(Strand=Plus/Minus) |
| *Kukri_c14239_1995* | No | *Tdurum_contig76595_208* | chr2A:7545332-7545232(Strand=Plus/Minus) |
| *Excalibur_c11587_340* | chr2B:616481860-616481760(Strand=Plus/Minus) | *wsnp_Ra_c21104_30458226* | chr2A:5677498-5677298(Strand=Plus/Minus) |
| *BS00012071_51* | chr2B:635023634-635023734(Strand=Plus/Plus) | *Ra_c510_171* | chr2A:5912422 -5912322(Strand=Plus/Minus) |
| *Excalibur_c74466_344* | No | *RAC875_c510_923* | chr2A:5911670-5911570(Strand=Plus/Minus) |
| *IAAV3305* | chr2B:126731379-126731509(Strand=Plus/Plus) | *tplb0032i02_1435* | chr2A:5677348-5677448(Strand=Plus/Plus) |
| *Kukri_rep_c109397_59* | chr5B:20667633-20667733(Strand=Plus/Plus) | *BS00068050_51* | chr2A:7514110-7514010(Strand=Plus/Minus) |
| *Excalibur_c3948_1315* | chr5B:19438843-19438743(Strand=Plus/Minus) | *BS00022242_51* | chr3B:42340498-42340598(Strand=Plus/Plus) |
| *Kukri_c2514_490* | chr5B:19439117-19439017(Strand=Plus/Minus) | *Excalibur_c25678_337* | chr3B:591455305-591455240(Strand=Plus/Minus) |
| *Kukri_c2514_583* | chr5B:19439024-19438924(Strand=Plus/Minus) | *Kukri_c7087_896* | chr3B:592610040-592610131(Strand=Plus/Plus) |
| *Tdurum_contig25432_1020* | chr5B:19439098-19439198(Strand=Plus/Plus) | *TA001464-0572* | chr3B:555738213-555738161(Strand=Plus/Minus) |
| *Tdurum_contig25432_1218* | chr5B:19439296-19439396(Strand=Plus/Plus) | *RAC875_c35801_905* | chr3D:438618143-438618073(Strand=Plus/Minus) |
| *Tdurum_contig25432_1377* | chr5B:19439455-19439555(Strand=Plus/Plus) | *CAP7_c4800_276* | chr5A:564497830-564497930(Strand=Plus/Plus) |
| *BobWhite_c3073_1156* | chr6A:5326517-5326417(Strand=Plus/Minus) | *IAAV8455* | chr5B:679559210-679559013(Strand=Plus/Minus) |
| *wsnp_Ku_c39334_47795350* | chr6A:5873055-5872855(Strand=Plus/Minus) | *BS00099729_51* | chr5B:703173238-703173138(Strand=Plus/Minus) |
| *wsnp_Ku_c39334_47795461* | chr6A:5872944-5872745(Strand=Plus/Minus) | *RAC875_c68525_284* | chr6B:657946476-657946576(Strand=Plus/Plus) |
| *BS00090253_51* | chr6A:6735208-6735108(Strand=Plus/Minus) | *Kukri_c4143_1055* | chr7B:594418349-594418276(Strand=Plus/Minus) |
| *Excalibur_c431_1130* | chr6A:5326475-5326375(Strand=Plus/Minus) | *RAC875_c18043_369* | chr7B:594418549-594418449(Strand=Plus/Minus) |
| *RAC875_c68978_220* | chr6A:5326296-5326398(Strand=Plus/Plus) | *RAC875_c18043_411* | chr7B:594418507-594418427(Strand=Plus/Minus) |
| *TA005787-0140* | chr6A:5363900-5363953(Strand=Plus/Plus) | *RAC875_c5646_774* | chr7B:594411384-594411284(Strand=Plus/Minus) |
