# Supplementary material for: Genome-wide association analysis of type II resistance to Fusarium head blight in common wheat
Source: PeerJ. 2023 Sep 21;11:e15906. doi: 10.7717/peerj.15906 (PMC10518165; doi:10.7717/peerj.15906)
Supplement: Table S3 [file peerj-11-15906-s003.docx]

**Supplementary Table 3** Functional prediction of candidate genes relate to wheat scab resistance

| Marker | Chromosome | Candidate gene | Molecular function | Biological process/Expression | Species |
| --- | --- | --- | --- | --- | --- |
| *RAC875_c35801_905* | 3DL | *TraesCS3D02G326700* | actin binding | actin filament organization  regulation of stomatal movement | Triticum aestivum |
|  |  | *TRIUR3_12331* | GTPase activity |  | Triticum urartu |
|  |  | *BGIOSGA033186* | ATP binding/  protein kinase activity |  | Oryza sativa Indica |
|  |  | *BGIOSGA031102* | UDP-glycosyltransferase activity |  |  |
|  |  | *Os06g0282400* | UDP-glycosyltransferase activity |  | Oryza sativa Japonica |
|  |  | *Os01g0888600（OsMLO5）* | calmodulin binding | defense response  response to biotic stimulus |  |
|  |  | *Os02g0564000（OsGSTZ3）* | glutathione transferase activity  maleylacetoacetate isomerase activity | glutathione metabolic process  L-phenylalanine catabolic process |  |
|  |  | *Os05g0500500* |  | Stress response |  |
|  |  | *Os08g0436400（OsSAP12）* | DNA binding  zinc ion binding | Stress response |  |
|  |  | *ORUFI10G15090* | ATP binding  protein kinase activity |  | Oryza rufipogon |
|  |  | *ORUFI06G11480* | UDP-glycosyltransferase activity |  |  |
|  |  | *ORUFI09G17870* | UDP-glycosyltransferase activity |  |  |
|  |  | *ORUFI08G04590* | UDP-glycosyltransferase activity |  |  |
|  |  | *ORUFI08G17770* | DNA binding/  zinc ion binding | Stress response |  |
| *D_contig74317_533* | 5DS | *TraesCS5D02G006700* | intramolecular transferase activity |  | Triticum aestivum |
|  |  | *HORVU1Hr1G083510* |  | defense response | Hordeum vulgare |
|  |  | *HORVU5Hr1G098670* |  | defense response |  |
|  |  | *HORVU4Hr1G063690* |  | defense response |  |
|  |  | *HORVU7Hr1G111700* | ADP binding/ATP binding | defense response |  |
|  |  | *HORVU7Hr1G100250* | ADP binding/ATP binding | defense response |  |
|  |  | *HORVU4Hr1G063910* | ATP binding/ chromatin binding | regulation of defense response |  |
|  |  | *HORVU7Hr1G020660* |  | calcium ion binding |  |
|  |  | *HORVU3Hr1G000980* | calcium ion binding/ ATP binding | protein phosphorylation |  |
|  |  | *HORVU3Hr1G111040* | ATP binding/ protein kinase activity | protein autophosphorylation |  |
|  |  | *AT3G55450(PBL1)* | nucleotide binding | defense response | Arabidopsis thaliana |
|  |  | *AT2G39510* | L-glutamine transmembrane transporter activity | L-glutamine import across plasma membrane |  |
|  |  | *BGIOSGA024654* | ATP-binding/ nucleotide binding | defense response | Oryza sativa Indica |
|  |  | *Os04g0413500(GIF1)* |  | [defense response to fungus](https://www.ebi.ac.uk/QuickGO/term/GO:0050832) | Oryza sativa Japonica |
|  |  | *ORUFI07G01690* | the defense reaction of plants against pathogens | [defense response](https://www.ebi.ac.uk/QuickGO/term/GO:0006952) | Oryza rufipogon |
|  |  | *ORUFI11G18220* |  | [toxin activity](https://www.ebi.ac.uk/QuickGO/term/GO:0090729)  [defense response](https://www.ebi.ac.uk/QuickGO/term/GO:0006952) |  |
| *IAAV9150* | 6AS | *TraesCS6A02G013700* | misfolded protein binding  polyubiquitin modification-dependent protein binding | ubiquitin-dependent ERAD pathway | Triticum aestivum |
|  |  | *TraesCS6A02G013800* | misfolded protein binding  polyubiquitin modification-dependent protein binding | ubiquitin-dependent ERAD pathway |  |
|  |  | *HORVU6Hr1G002550* | GTP binding |  | Hordeum vulgare |
|  |  | *AT1G24010* |  | defense response | Arabidopsis thaliana |
|  |  | *AT5G43590* | transferase activity | [defense response](https://www.ebi.ac.uk/QuickGO/term/GO:0006952) |  |
|  |  | *AT1G22870* | ATP binding/  protein kinase activity |  |  |
|  |  | *AT1G05810（ARA）* | GTPase activity  GTP binding |  |  |
|  |  | *AT3G56770（BHLH107）* | DNA binding  DNA-binding transcription factor activity  protein dimerization activity | regulation of transcription, DNA-templated  response to chitin |  |
|  |  | *BGIOSGA008772* | ATP binding  protein kinase activity |  | Oryza sativa Indica |
| *Excalibur_c20597_509* | 6AS | *TraesCS6A02G013600* | GTP binding |  | Triticum aestivum |
|  |  | *HORVU2Hr1G001560* | ATP binding  protein kinase activity |  | Hordeum vulgare |
|  |  | *HORVU6Hr1G028220* | ADP binding  ATP binding | defense response |  |
|  |  | *HORVU3Hr1G105190* | glucan endo-1,3-beta-D-glucosidase activity | carbohydrate metabolic process  defense response |  |
|  |  | *HORVU3Hr1G021810* | UDP-glycosyltransferase activity |  |  |
|  |  | *HORVU6Hr1G070200* | UDP-glycosyltransferase activity |  |  |
|  |  | *HORVU5Hr1G008990* | calcium ion binding  protein serine/threonine kinase activity |  |  |
|  |  | *HORVU5Hr1G106720* | calcium ion binding  polysaccharide binding  protein serine/threonine kinase activity | cell surface receptor signaling pathway  protein phosphorylation |  |
|  |  | *AT2G22805* |  | defense response to fungus/killing of cells of other organism | Arabidopsis thaliana |
|  |  | *AT1G51960（IQD27）* | calmodulin binding |  |  |
|  |  | *AT5G63660（PDF2.5）* |  | defense response  defense response to fungus  killing of cells of other organism |  |
|  |  | *AT1G17910（WAKL13）* | ATP binding  polysaccharide binding  protein serine/threonine kinase activity | cell surface receptor signaling pathway  protein phosphorylation |  |
|  |  | *AT3G53840（WAKL15）* | ATP binding  protein serine/threonine kinase activity | cell surface receptor signaling pathway  protein phosphorylation |  |
|  |  | *AT1G18670（IBS1）* | ATP binding  protein kinase activity |  |  |
|  |  | *AT1G77630（LYM3）* | peptidoglycan binding | defense response  immune response |  |
|  |  | *BGIOSGA032454* | ATP binding  calcium ion binding polysaccharide binding  protein serine/threonine kinase activity |  | Oryza sativa Indica |
|  |  | *BGIOSGA032260* | ATP binding  calcium ion binding polysaccharide binding  protein serine/threonine kinase activity |  |  |
|  |  | *BGIOSGA032460* | ATP binding  calcium ion binding  polysaccharide binding  protein kinase activity |  |  |
|  |  | *BGIOSGA020567* | ATP binding  calcium ion binding polysaccharide binding  protein kinase activity |  |  |
|  |  | *BGIOSGA032540* | calcium ion binding polysaccharide binding  protein serine/threonine kinase activity |  |  |
|  |  | *BGIOSGA020636* | calcium ion binding  polysaccharide binding  protein serine/threonine kinase activity |  |  |
|  |  | *BGIOSGA032280* | calcium ion binding polysaccharide binding  protein serine/threonine kinase activity |  |  |
|  |  | *BGIOSGA037795* | calcium ion binding  polysaccharide binding |  |  |
|  |  | *BGIOSGA030347* | ATP binding  protein kinase activity |  |  |
|  |  | *BGIOSGA015265* | calcium ion binding |  |  |
|  |  | *BGIOSGA007337* | GTP binding |  |  |
|  |  | *Os04g0220300（OsWAK30）* | calcium ion binding  polysaccharide binding  protein serine/threonine kinase activity |  | Oryza sativa Japonica |
|  |  | *Os07g0493800（OsWAK71）* | ATP binding  protein serine/threonine kinase activity | cell surface receptor signaling pathway  protein phosphorylation |  |
|  |  | *Os10g0151100（OsWAK103）* | ATP binding  calcium ion binding  polysaccharide binding  protein serine/threonine kinase activity | cell surface receptor signaling pathway  protein phosphorylation |  |
|  |  | *Os10g0151500（OsWAK104）* | ATP binding  calcium ion binding polysaccharide binding  protein serine/threonine kinase activity | cell surface receptor signaling pathway  protein phosphorylation |  |
|  |  | *Os10g0152000（OsWAK105）* | ATP binding  calcium ion binding polysaccharide binding  protein serine/threonine kinase activity | cell surface receptor signaling pathway  protein phosphorylation |  |
|  |  | *Os10g0326200（OsWAK114）* | ATP binding  protein serine/threonine kinase activity | cell surface receptor signaling pathway  protein phosphorylation |  |
|  |  | *Os12g0615300（OsWAK129）* | ATP binding  calcium ion binding  polysaccharide binding  protein serine/threonine kinase activity | cell surface receptor signaling pathway  protein phosphorylation |  |
|  |  | *Os03g0642600（OsRLCK114）* | ATP binding | cell surface receptor signaling pathway |  |
|  |  |  | polysaccharide binding | protein phosphorylation |  |
|  |  |  | protein serine/threonine kinase activity |  |  |
|  |  | *Os04g0371200（OsRLCK148）* | ATP binding  protein serine/threonine kinase activity | cell surface receptor signaling pathway  protein phosphorylation |  |
|  |  | *Os07g0494800（OsRLCK231）* | ATP binding  protein serine/threonine kinase activity | cell surface receptor signaling pathway  protein phosphorylation |  |
|  |  | *Os11g0691280（OsRLCK353）* | ATP binding  calcium ion binding polysaccharide binding  protein serine/threonine kinase activity | cell surface receptor signaling pathway  protein phosphorylation |  |
|  |  | *Os11g0432900（OsSCP56）* | serine-type carboxypeptidase activity  transferase activity | secondary metabolic process |  |
|  |  | *Os02g0104700（NOA1）* | GTP binding  nitric-oxide synthase activity |  |  |
|  |  | *ORUFI04G05310* | ATP binding  calcium ion binding  polysaccharide binding  protein serine/threonine kinase activity |  | Oryza rufipogon |
|  |  | *ORUFI10G04150* | ATP binding  calcium ion binding polysaccharide binding  protein serine/threonine kinase activity |  |  |
|  |  | *ORUFI11G24870* | ATP binding  calcium ion binding  polysaccharide binding  protein serine/threonine kinase activity |  |  |
|  |  | *ORUFI12G20980* | ATP binding  calcium ion binding  polysaccharide binding  protein serine/threonine kinase activity |  |  |
|  |  | *ORUFI11G24960* | ATP binding  calcium ion binding  protein kinase activity |  |  |
|  |  | *ORUFI02G00430* | GTP binding |  |  |
| *BS00025286_51* | 7BL | *TraesCS7B02G370700* | chitinase activity | cell wall macromolecule catabolic process  chitin catabolic process  defense response to fungus  polysaccharide catabolic process | Triticum aestivum |
|  |  | *HORVU1Hr1G052430（chitinase）* | chitinase activity | cell wall macromolecule catabolic process  chitin catabolic process  polysaccharide catabolic process | Hordeum vulgare |
|  |  | *HORVU7Hr1G121850* | chitinase activity  chitin binding | cell wall macromolecule catabolic process  chitin catabolic process  defense response to fungus  polysaccharide catabolic process |  |
|  |  | *TRIUR3_19310* | chitinase activity | cell wall macromolecule catabolic process  chitin catabolic process  polysaccharide catabolic process | Triticum urartu |
|  |  | *BGIOSGA023632* | chitinase activity  chitin binding | cell wall macromolecule catabolic process  chitin catabolic process  defense response to fungus  polysaccharide catabolic process | Oryza sativa Indica |
|  |  | *BGIOSGA023633* | chitinase activity  chitin binding | cell wall macromolecule catabolic process  chitin catabolic process  polysaccharide catabolic process |  |
|  |  | *BGIOSGA026643* | chitinase activity | cell wall macromolecule catabolic process  chitin catabolic process  polysaccharide catabolic process |  |
|  |  | *Os06g0726100（Cht3*）* | beta-N-acetylglucosaminidase activity  chitinase activity  chitin binding  endochitinase activity | amino sugar metabolic process  cell wall macromolecule catabolic process  chitin catabolic process  defense response to fungus  polysaccharide catabolic process | Oryza sativa Japonica |
|  |  | *Os06g0726200(Chi1)* | chitinase activity  chitin binding | cell wall macromolecule catabolic process  chitin catabolic process  defense response to fungus  polysaccharide catabolic process |  |
|  |  | *Os02g0605900（Cht6）* | chitinase activity  chitin binding | cell wall macromolecule catabolic process  chitin catabolic process  defense response  polysaccharide catabolic process |  |
|  |  | *Os08g0522500* | chitinase activity | cell wall macromolecule catabolic process  chitin catabolic process  polysaccharide catabolic process |  |
| *Kukri_c4143_1055* | 7BL | *TraesCS7B01G340200* | EH domain-containing protein 1 |  | Triticum aestivum |
|  |  | *HORVU4Hr1G003110* | G-protein gamma-subunit binding | defense response to fungus, incompatible interaction  G protein-coupled receptor signaling pathway  jasmonic acid mediated signaling pathway  response to extracellular stimulus | Hordeum vulgare |
|  |  | *HORVU3Hr1G113790* | ADP binding  ATP binding | defense response |  |
|  |  | *HORVU4Hr1G000350* | ADP binding  ATP binding | defense response |  |
|  |  | *HORVU6Hr1G093820* | ATP binding  calcium ion binding  protein serine/threonine kinase activity |  |  |
|  |  | *HORVU5Hr1G104080* | ATP binding  protein kinase activity |  |  |
|  |  | *HORVU4Hr1G029350* | ATP binding  protein kinase activity | protein-chromophore linkage  regulation of transcription, DNA-templated  response to stimulus |  |
|  |  | *HORVU5Hr1G110920* |  | defense response |  |
|  |  | *HORVU6Hr1G083620* | diacylglycerol kinase activity  NAD+ kinase activity | defense response  protein kinase C-activating G protein-coupled receptor signaling pathway |  |
|  |  | *TRIUR3_24813* | calcium ion binding  GTP binding |  | Triticum urartu |
|  |  | *AT5G46470（RPS6）* | ADP binding  NAD(P)+ nucleosidase activity  NAD+ nucleotidase, cyclic ADP-ribose generating | defense response  signal transduction | Arabidopsis thaliana |
|  |  | *AT3G06730（CITRX）* | disulfide oxidoreductase activity Source: GO_Central  protein disulfide oxidoreductase activity Source: TAIR  protein-disulfide reductase activity | cell redox homeostasis  defense response  plastid organization |  |
|  |  | *AT2G01520（MLP328）* | copper ion binding | defense response |  |
|  |  | *BGIOSGA002446* | calcium ion binding |  | Oryza sativa Indica |
|  |  | *Os06g0687800* | calcium ion binding  GTP binding |  | Oryza sativa Japonica |
|  |  | *Os01g0135700（OsCML16）* | calcium ion binding |  |  |
|  |  | *ORUFI01G22930* | ATP binding  protein kinase activity |  | Oryza rufipogon |
|  |  | *ORUFI04G30660* | calcium ion binding  GTP binding |  |  |
|  |  | *ORUFI06G27260* | calcium ion binding  GTP binding |  |  |
|  |  | *ORUFI01G02360* | calcium ion binding |  |  |
| *RAC875_c5646_774* | 7BL | *TraesCS7B02G340100* | galactosylgalactosylxylosylprotein 3-beta-glucuronosyltransferase activit  xylosyltransferase activity | carbohydrate metabolic process  cell wall organization  glucuronoxylan biosynthetic process  plant-type secondary cell wall biogenesis | Triticum aestivum |
|  |  | *HORVU2Hr1G036030* | protein serine/threonine kinase activator activity | intracellular signal transduction | Hordeum vulgare |
|  |  | *AT1G79620* | ATP binding  protein kinase activity  protein serine/threonine kinase activity |  | Arabidopsis thaliana |
|  |  | *BGIOSGA017394* | ATP binding  protein kinase activity |  | Oryza sativa Indica |
|  |  | *BGIOSGA010030* | actin binding  ATP binding  calmodulin binding  motor activity | actin filament organization |  |
|  |  | *Os04g0517500* | ATP binding  protein serine/threonine kinase activity |  | Oryza sativa Japonica |
|  |  | *ORUFI12G13460* | ADP binding  ATP binding | defense response | Oryza rufipogon |
|  |  | *ORUFI01G37780* | ADP binding  ATP binding | defense response |  |
|  |  | *ORUFI04G32270* | ATP binding  protein kinase activity |  |  |
