# Supplementary material for: Genome-wide association analysis of type II resistance to Fusarium head blight in common wheat
Source: PeerJ. 2023 Sep 21;11:e15906. doi: 10.7717/peerj.15906 (PMC10518165; doi:10.7717/peerj.15906)
Supplement: Table S4 — “+” represents the allele for improving scab resistance; “-” represents the allele that reduces the resistance [file peerj-11-15906-s004.docx]

**Supplementary Table 4** Plant height statistics of different resistant locus

| Locus | Chromosome | Allele | Variety  number | Mean of Plant height | P-value |
| --- | --- | --- | --- | --- | --- |
| *D_contig74317_533* | 5D | + | 28 | 73.08 | 1.65E-04 |
|  |  | - | 177 | 73.57 |  |
| *Kukri_c14239_1995* | 1B | + | 11 | 73.80 | 9.70E-05 |
|  |  | - | 192 | 73.49 |  |
| *BS00025286_51* | 7B | + | 52 | 75.00 | 2.84E-05 |
|  |  | - | 125 | 72.58 |  |
| *RAC875_c35801_905* | 3D | + | 19 | 71.57 | 3.72E-05 |
|  |  | - | 186 | 73.70 |  |
| *Kukri_c4143_1055* | 7B | + | 101 | 73.16 | 2.49E-04 |
|  |  | - | 104 | 73.83 |  |

**“+”** represents the allele for improving scab resistance; **“-”** represents the allele that reduces the resistance
