# Supplementary material for: Genome-wide association analysis of type II resistance to Fusarium head blight in common wheat
Source: PeerJ. 2023 Sep 21;11:e15906. doi: 10.7717/peerj.15906 (PMC10518165; doi:10.7717/peerj.15906)
Supplement: Table S5 — “+” represents the allele for improving scab resistance; “-” represents the allele that reduces the resistance [file peerj-11-15906-s005.docx]

**Supplementary Table 5** Plant height statistics of different resistant QTL combinations

| Genotype | *D_contig74317_533* | *Kukri_c14239_1995* | *BS00025286_51* | *RAC875_c35801_905* | *Kukri_c4143_1055* | Variety  number | Mean of Plant height | Effect of QTL combinations | Standard deviation | *F*-value | *P*-value |
| --- | --- | --- | --- | --- | --- | --- | --- | --- | --- | --- | --- |
|  | - | - | - | - | - | 38 | 75.2 | . | 1.83 | 1.924 | 0.016 |
|  | + | - | - | - | - | 9 | 69.9 | -0.070 | 2.20 |  |  |
|  | - | + | - | - | - | 3 | 70.1 | -0.068 | 3.300 |  |  |
|  | - | - | + | - | - | 28 | 72.1 | -0.041 | 1.14 |  |  |
|  | - | - | - | + | - | 2 | 75.0 | -0.003 | 1.05 |  |  |
|  | - | - | - | - | + | 62 | 71.4 | -0.051 | 0.51 |  |  |
|  | + | - | + | - | - | 1 | 67.8 | -0.098 | . |  |  |
|  | + | - | - | - | + | 1 | 91.5 | 0.016 | . |  |  |
|  | - | - | + | + | - | 2 | 73.0 | -0.029 | 2.45 |  |  |
|  | - | - | + | - | + | 10 | 82.4 | 0.096 | 4.70 |  |  |
|  | - | - | - | + | + | 2 | 71.2 | -0.053 | 0.10 |  |  |
|  | + | + | - | + | - | 2 | 69.7 | -0.073 | 7.20 |  |  |
|  | + | + | - | - | + | 1 | 83.9 | 0.116 | . |  |  |
|  | + | - | + | - | + | 2 | 86.9 | 0.156 | 1.99 |  |  |
|  | + | - | - | + | + | 3 | 68.3 | -0.092 | 2.62 |  |  |
|  | - | + | + | + | - | 2 | 73.5 | -0.023 | 2.45 |  |  |
|  | - | + | + | - | + | 1 | 74.4 | -0.011 | . |  |  |
|  | - | - | + | + | + | 3 | 72.5 | -0.036 | 0.20 |  |  |
|  | + | + | + | - | + | 1 | 79.5 | 0.057 | . |  |  |
|  | + | - | + | + | + | 1 | 75.4 | 0.003 | . |  |  |

**“+”** represents the allele for improving scab resistance; **“-”** represents the allele that reduces the resistance
